# Preliminary use of a double-echo pulse sequence with 3D ultrashort echo time in the MRI of bones and joints

**DOI:** 10.3892/etm.2013.993

**Published:** 2013-03-07

**Authors:** LIHENG MA, QUANFEI MENG, YINGMING CHEN, ZHAOHUI ZHANG, HAIXING SUN, DEMAO DENG

**Affiliations:** 1 The Medical Diagnostic Center, The Sixth Affiliated Hospital of Sun Yat-Sen University, Guangzhou, Guangdong 510080;; 2 Department of Radiology, The First Affiliated Hospital of Sun Yat-Sen University, Guangzhou, Guangdong 510080;; 3 Department of Radiology, The First Hospital of Traditional Chinese Medical University, Nanning, Guangxi 530023, P.R. China

**Keywords:** bone, joint, magnetic resonance imaging, ultrashort echo time, pulse sequence

## Abstract

The aim of the present study was to investigate the application of a double-echo pulse sequence with 3D ultrashort echo time (UTE) in the magnetic resonance imaging (MRI) of bones and joints. In total, 7 healthy volunteers and 1 volunteer with a suspected tear of the lateral meniscus of the left knee joint underwent MRI with a double-echo pulse sequence and 3D UTE. The imaging was performed on the tibial diaphysis, knee joint and ankle of the volunteers and on a segment of porcine fibula *in vitro*. The echo time of echo 1 (TE1) of the UTE images for the achilles tendon of the ankle joint were set as 0.08, 0.16, 0.24 and 0.35 msec. The maximum intensity projection (MIP) of the difference images created from the primary double-echo images with a TE1 of 0.08 msec were performed on the tendons of the ankle to display their 3D structure. The data were analyzed with a one-way ANOVA and paired-sample t-test. The 3D distribution of the tendons was displayed through MIPs of the difference images created from the primary double-echo images. The cortical bones, periosteum, tendons and menisci of the 8 volunteers appeared as high signal intensities in the UTE pulse sequence. Multiplanar reconstruction followed by subtraction of the primary double-echo images raised the image signal-to-noise (S/N) ratio from 2.80±0.75 to 3.76±0.88 (t=−4.851, P<0.01). The artifacts appeared more marked as the TE1 was prolonged. A double pulse sequence MRI with 3D UTE may display the short T_2_ components which are not displayed with a conventional clinical MRI sequence, therefore creating a basis for the further quantification of these tissues.

## Introduction

The most commonly used method for diagnosising parenchymal disease with traditional clinical magnetic resonance imaging (MRI) is the detection of the long-transverse relaxation time (T_2_) signal in normal or diseased tissues. The T_2_ in certain tissues of the human body is very short, which would lead to the extremely quick decay of MR signals derived from these short-T_2_ tissues in conventional MR sequence imaging. The MR signals would decay to 0 or near 0 prior to the clinical MRI system entering the signal receiving mode. Therefore, these organs would show low or no signal in conventional MRI (1). A low or absent signal creates a difficulty in making an image appear using various pulse sequences or contrast agents, making quantitative determination impossible.

Several methods have been developed for the imaging of short-T_2_ components (2–4). With the breakthrough in imaging methods, the following possibilities have been raised with regard to the clinical applications of MRI: i) if the acquisition time window was shortened, which would involve lowering the imaging T_2_ window level and zooming out the displaying-window width towards the imaging of the short-T_2_ components or tissues, then the signals of the short-T_2_ components may be collected and varying short-T_2_ tissues may be distinguished even by MRI; ii) further development of the application horizontally to enable expansion of the clinical window range; and iii) by collecting the signals of the short-T_2_ components, further quantitative studies of these tissues and organs may be performed.

Based on these issues, the present study used a double-echo pulse sequence with 3D ultrashort echo time (UTE) for the imaging of short-T_2_ components and a series of research procedures. Double pulse sequence MRI with 3D UTE was performed on the tibial diaphysis, knee and ankle of a group of normal individuals and on 1 case of a suspected tear of the left knee meniscus to investigate the application of an imaging sequence in the bones and joints.

## Materials and methods

### General information

In total, 7 healthy volunteers and 1 with a suspected tear of the lateral meniscus of the left knee joint were included in the present study. Their ages ranged between 31 and 46 years old, with a median age of 38 years old. Of these patients, 6 were male aged between 31 and 46 years old (median, 37 years) and 2 were female aged 38 and 39 years old. The present study was conducted in accordance with the Declaration of Helsinki and with approval from the Ethics Committee of the First Affiliated Hospital of Sun Yat-Sen University (Guangzhou, China). Written informed consent was obtained from all participants. A double-echo pulse sequence MRI with 3D UTE was performed on the tibial diaphysis, knee joint and ankle.

In addition, the same MRI was performed on a segment of non-muscle-stripped porcine fibula *in vitro*.

### Imaging equipment and the pulse sequence

**Figure 1:**
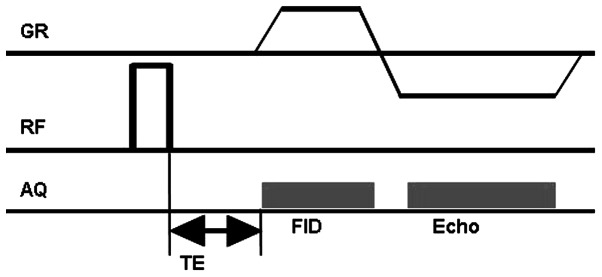
Following a block hard pulse (<100 *μ*sec) and a delay time, the 1st ultrashort echo (echo 1), which was generated by the free induction decay signal of the tissue during gradient slope-rising and the plateau phase, was recorded. Subsequently, the 2nd echo signal (echo 2) was generated with a reversed phase. GR, gradient; RF, radio frequency; AQ, acquisition; TE, echo time; FID, free induction decay.

A 1.5-T MR imaging system (Intera Achieva, Philips Healthcare, Guangzhou, China) and Flex-M surface coil were used in the imaging process. The pulse sequence was a dual-echo pulse sequence with 3D UTE. Following a block hard pulse (a type of radio frequency pulse; <100 *μ*sec) and a delay time, the 1st ultrashort echo (echo 1), which was generated by the free induction decay signal of the tissue during gradient slope-rising and plateau phase, was recorded. Subsequently, the 2nd echo signal (echo 2) was generated with a reversed phase (the sequence design is shown in Fig. 1). The fill of the k-space was created by the 3D isotropic radial trajectory starting from the k-space center. The switching time of the coil was used to determine the shortest echo time of echo 1 (TE1), which may be <100 *μ*sec. The switching time of the Flex-M surface coil was <30 *μ*sec. Combined with other acquisition parameters, the shortest TE1 used was 80 *μ*sec and the TE of echo 2 (TE2) was 4.6 msec (the inphase time of water and fat under 1.5 T).

### Scan program and parameters

**Figure 2:**
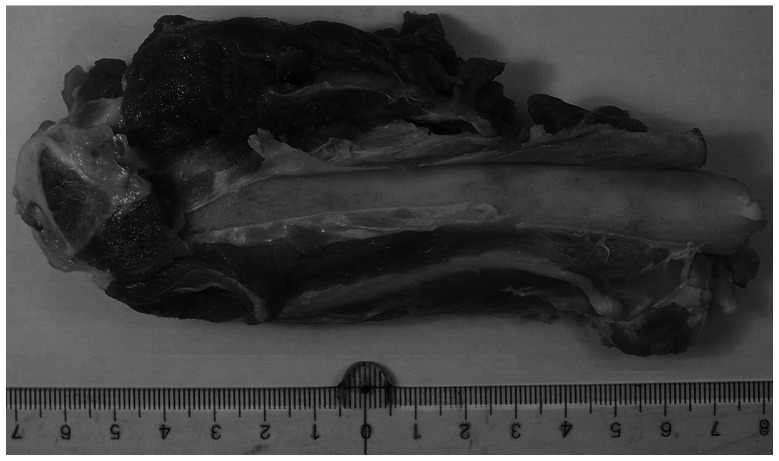
Non-muscle-stripped porcine fibula. The muscles were separated bluntly and part of the periosteum was stripped and freed while still being connected to the non-stripped periosteum. The free periosteal edge was then curled outwardly.

A 3D UTE double-echo pulse sequence MRI was performed to gain the 3 sagital, coronal and axial planar images of the volunteers’ tibial diaphysis, knee and ankle and of the non-muscle-stripped porcine fibula *in vitro*. To investigate the influence of TE1 on the image quality, 3D UTE double-echo pulse sequence imaging with TE1s of 0.08, 0.16, 0.24 and 0.35 msec were performed on the ankles and achilles tendons of the volunteers. Following the 1st imaging of the non-muscle-stripped porcine fibula, the partial muscles were detached bluntly, the periosteum was stipped partially while still connected with the non-stripped periosteum, then the free periosteal edge was curled outwardly (Fig. 2) and the MR double-echo pulse sequence imaging with 3D UTE was performed again.

The imaging parameters were as follows: TR, 7.9–9.3 msec; TE1, 0.08–0.35 msec; TE2, 2.3–4.6 msec; field of view (FOV), 70×70×70–140×140×140 mm; matrix, 88×88–124×124mm; acquisition voxel, 0.8×0.8×0.8–1.21×1.21×1.21 mm; reconstruction voxel, 0.63×0.63×0.63 mm; density of angle, 75%; flip angle, 8 and 12°; and a trajectory delay time of 1 and 2 *μ*sec .

### Post-processing of the images

To highlight the short T_2_ components in the tissues, the 2nd echo sequence images were subtracted from the 1st echo sequence images to remove or reduce the signals from the long-T_2_ components in the tissues and the difference images mainly containing short-T_2_ components were obtained. In addition, multiplanar reconstructions (reconstruction thickness, 2 mm) of the 3D imaging data of every echo were performed, then the 2nd echo multiplanar reconstruction images were subtracted from the 1st echo multiplanar reconstruction images and the difference images with a short-T_2_ component signal were obtained. For the ankle tendon imaging, subsequent to subtracting the primary 2nd echo sequence images from the primary 1st echo sequence images, 3D images of the ankle tendon were obtained when the 3D reconstructions (reconstruction thickness, 2 mm) were performed on the difference images with the maximum intensity projection (MIP) method.

To investigate the impact of various TE1s on image quality, the images were compared and analyzed (mainly with regard to the quantity of the artifacts). A rectangular region of interest was used to measure the signal intensity of the achilles tendon and bone marrow and background signal intensity of the anterior distal tibial diaphysis in the 4 TE1s of the primary echo difference images of the ankle. The contrast-to-noise ratio was also calculated using the signal intensity (SI) using the formula SI_tendon_ - SI_marrow cavity_ / SI_background_. The size of the region of interest was 10 mm^2^ for the achilles tendon, 100 mm^2^ for the bone marrow and 200 mm^2^ for the background. At the same time, the rectangular region of interest was used to measure the SI of the achilles tendon and the anterior background SI of the distal tibial diaphysis in the TE1 0.08 msec primary echo difference and multiplanar reconstruction difference images for the calculation of the signal-to-noise ratio (SNR).

### Statistical analysis

SPSS 13.0 statistical software was used for the statistical analysis of the differences in the contrast-to-noise ratio of the achilles tendon and bone marrow in the primary echo difference images of the various TE1s, as well as for the signal-to-noise (S/N) ratio of TE1 0.08 msec primary echo and multiplanar reconstruction difference images. The statistical methods used were a one-way ANOVA (pairwise comparisons using the Student-Newman-Keuls-q test) and paired sample t-test. P<0.05 was considered to indicate a statistically significant result.

## Results

### Signal characteristics of the cortical bone and periosteum in the UTE imaging sequence

**Figure 3:**
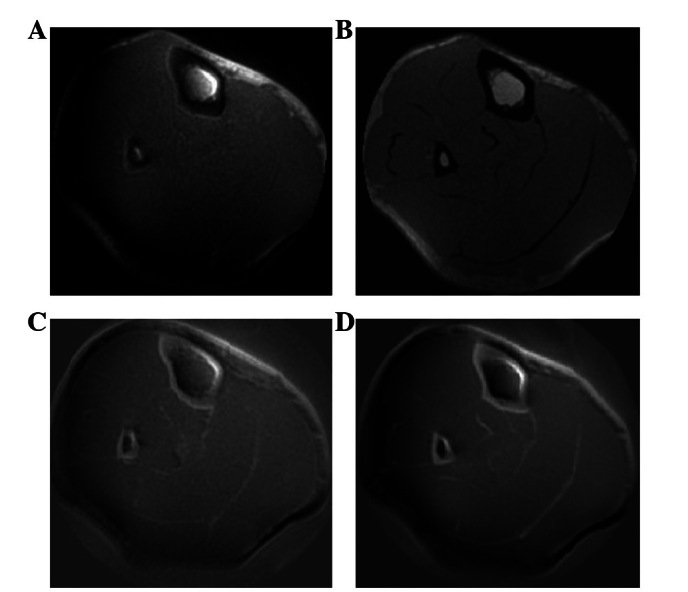
(A) 1st echo image of the ultrashort echo time (UTE) sequence. The tibial cortical bone was exhibited as a slight hyperintensity, while the periosteum was exhibited as a linear hyperintensity around the cortical bone. (B) The 2nd echo image of the UTE sequence. The tibial cortical bone was exhibited as a low signal intensity, while the periosteum was unrecognizable due to decay. (C) The difference image subtracted from the original 1st and 2nd echo images. The tibial cortical bone was exhibited as a hyperintensity, the periosteum with a partial hyperintensity remained observable and the muscular fasciae was exhibited as a hyperintensity. (D) The difference image obtained from the subtraction of the original echoes reconstructed by the multiplanar reconstruction. It was observable that the signal-to-noise (S/N) ratio was higher than the direct subtraction of the two original echoes.

The tibial cortical bone of the 8 subjects was exhibited as a slight hyperintensity on the 1st echo sequence, while the periosteum was exhibited as a linear hyperintensity around the cortical bone. In the 2nd echo sequence, the cortical bone was exhibited as a low signal intensity, while the periosteum appeared unclear. Following the subtraction of the two echo sequence images, the cortical bone appeared as a significant hyperintensity with a clear outline and the periosteum was exhibited as a partially visible hyperintensity around the cortical bone (Fig. 3A–C). The S/N ratio was higher in the difference images of the multiplanar reconstruction than in the primary echo difference images (Fig. 3C and D).

**Figure 4:**
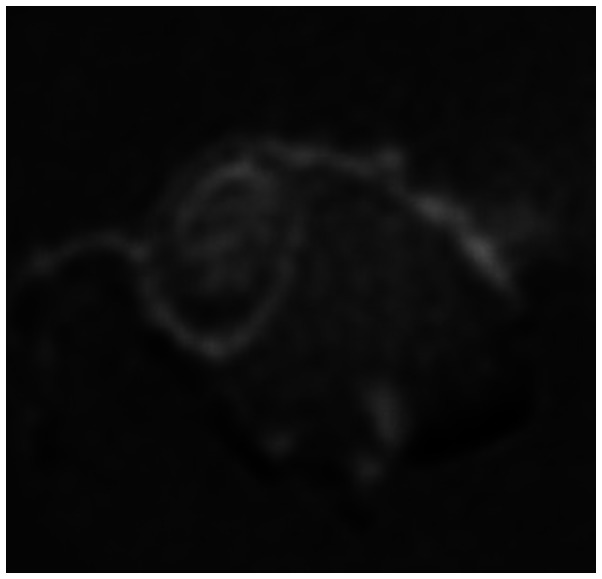
Difference image obtained from the multiplanar reconstruction of the ultrashort echo time (UTE) imaging of the partially-stripped and outwardly curling periosteum which was connected with the non-muscle-stripped periosteum in the porcine rear fibula.

The periosteum of the non-periosteum-stripped porcine fibula exhibited a linear hyperintensity around the cortical bone in the 1st echo image and an unclear signal in the 2nd echo image, while in the difference images of the 2 echo subtractions, the partial hyperintensity of the periosteum remained observable. The stripped- and freed-partial-periosteum porcine fibula connected with the periosteum which had not been stripped from the cortical bone exhibited a free and outwardly curling linear hyperintensity (Fig. 4).

### Meniscal signal characteristics of the knee joint

**Figure 5:**
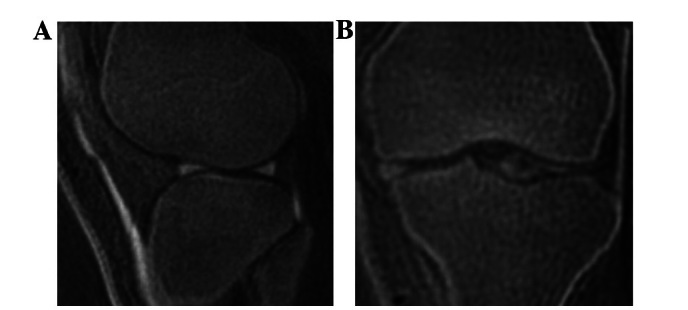
(A) Difference images obtained from the multiplanar reconstruction of a normal volunteer. The knee meniscus was exhibited as a hyperintensity, the rear section of the posterior angle of the posterior cruciate ligament of the lateral meniscus also showed as a hyperintensity and the patellar ligament was a clearly observable hyperintensity. (B) Torn meniscal fragments with a visible hyperintensity were observed shifting to the femoral intercondylar fossa.

The meniscus exhibited a hyperintensity in the difference images following echo subtraction (Fig. 5). The torn meniscal fragments were observed as a visible hyperintensity shifting to the femoral intercondylar fossa in the difference images of the volunteer with the clinically suspected lateral meniscus tear (Fig. 5B).

### Signal characteristics of the ligament in the UTE pulse sequence

**Figure 6:**
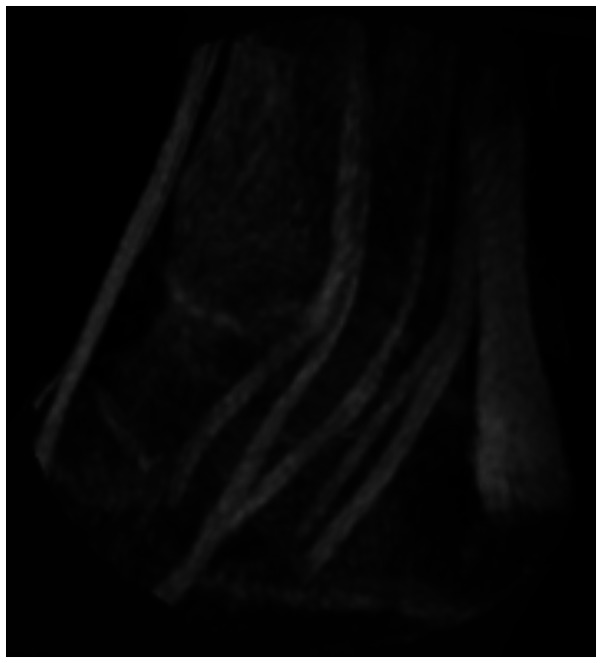
3D information of the spatial distribution and direction of the ligaments were obtained from 3D images resulting from the 3D reconstruction of the difference images using the maximum intensity projection (MIP).

The UTE double-echo imaging of the ankle showed a slightly higher signal for the ligament around the joint in the 1st echo images and a low signal in the 2nd echo images, while a significantly higher signal was observed in the difference images following the subtraction of the two primary echo images. The adjacent long-T_2_ tissues presented low signals. The 3D information for the ligaments were obtained from the 3D images that resulted from the 3D reconstruction of the difference images using the MIP (Fig. 6).

### Effect of TE1 time and post-processing techniques on the images

The image analysis results showed that when comparing the various TE1s, the UTE image quality of 0.08 msec was the best; as the TE1 time was prolonged, the artifacts gradually increased. When the TE1s were 0.08, 0.16, 0.24 and 0.35 msec, the contrast-to-noise ratios were 1.74±0.54, 1.35±0.60, 1.2±0.48 and 0.89±0.24, respectively, with a statistically significant difference (F=3.681, P<0.05). Pairwise comparison results showed that the image contrast-to-noise ratio of a TE1 of 0.08 msec was higher than that of the other 3 TE1 images and exhibited statistically significant differences (q= 2.95, 4.08 and 6.43, respectively; P<0.05) while the other TE1 image contrast-to-noise ratios had no statistically significant differences among them (q=1.13, 2.34 and 3.48, respectively; P>0.05). The paired-sample t-test results demonstrated that the S/N ratio was higher in the difference images of the multiplanar reconstruction (3.76±0.88) than in the primary echo difference images (2.82±0.75) with a statistically significant difference (t=−4.851; P<0.01).

## Discussion

The most common approach for the diagnosis of parenchymal disease using MRI is the use of heavily T_2_-weighted pulse sequences to detect the signal from the long-T_2_ relaxation components in normal tissues and an increase or decrease in the signal from these components in any abnormal tissues. Even the diagnostic theories of the new pulse sequences, including fast spin-echo, echo-planar imaging and fluid-attenuated-inversion recovery sequences remain unable to detect the long-T_2_ signals in the normal or diseased tissues.

Henkelman *et al*(5) regarded tissues with T_2_ values of <10 msec as short-T_2_ values (in the light of more recent pulse sequence developments, this threshold requires downward adjustment). The water protons in a number of biological tissues have T_2_ relaxation times of <1 msec. Such extremely short-T_2_ values result from the restricted mobility of water when bound to collagen or confined to small spaces (6). The tissues with short-T_2_ components may be divided into tissues with a minority of short-T_2_ components and tissues with a majority of short-T_2_ components. Skeletal muscle is a tissue containing a minority of short-T_2_ components (7).

A series of extremely short T_2_ tissue imaging techniques have been reported in the past 20 years, including single point imaging technology, single point slope imaging with enhanced T_1_, multi-point imaging (2) and water and fat-suppressed MRI (3). It has been confirmed that single point imaging technology and its variants (including multi-point imaging) are particularly effective for displaying extremely short T_2_ components, while requiring a fast and extremely strong gradient and a scan time that is too long to be suitable for human imaging *in vivo*. Other technologies for detecting short-T_2_ components in the developmental and application stages are magnetization transfer imaging, magic angle imaging, short TE imaging and UTE pulse sequence imaging (4). The TE ranges of various pulse sequences are shown in Table I(8).

A UTE pulse sequence is designed to solve the difficulty in exciting the short-T_2_ components and rapidly collecting signals prior to signal decay. Bergin *et al*(9,10) first used the semi-radio frequency (RF) pulse and radial sampling pulse sequence clinically in lung imaging in 1991 and 1992. The sequence was then used for imaging other areas and its application in the musculoskeletal system began in 1995 (11–19). Since then, the UTE sequence has been more widely applied to the musculoskeletal system (20–24). The acronyms used to describe the UTE sequences are shown in Table II(25).

In the present study, a double-echo pulse sequence MRI with 3D UTE was used. In order to emphasize the short-T_2_ components, difference images were obtained by subtracting the 2nd echo images from the 1st echo images to remove or reduce the signal of the long-T_2_ components. Thus, the images that contained short-T_2_ components were displayed. The 3D UTE imaging was performed on the partially-freed-periosteum porcine fibula *in vitro* to confirm that the 3D UTE pulse sequence was able to define the periosteum which is not observable in conventional MRI. The cortical bone, menisci and ankle ligaments were shown as hyperintensities by the UTE sequence, making detection of these tissues containing the majority of the short-T_2_ components available using 2 types of methods, namely those of high and low signals, and increasing the information available for clinical diagnostic work.

Using 3D acquisition through the MIP, 3D UTE imaging may provide 3D information on tendons for clinics and new anatomical reference information for the clinical diagnosis and treatment of tendon injuries. In the present study, a 2-mm thick reconstruction of the two primary echo images was performed using a multiplanar reconstruction technique and the post-reconstruction difference images were obtained by echo subtraction to markedly improve the the S/N ratio of the images. For the achilles tendon imaging, the best image quality was obtained in a TE1 of 0.08 msec following a comparison of 4 TE times. Free induction decay signals were collected in the present study and the signal attenuation was fast, therefore a short TE1 time should be used in future imaging whenever possible.

In conclusion, a double pulse sequence MRI with 3D UTE may display the short-T_2_ components which are not displayed in conventional clinical MRI sequences. The sequence lowers and zooms out the T_2_ window of the tissues that may be displayed in MRI. The visualization of the tissues by MRI is extended, founding a basis for further quantification of these tissues.

Considering that the resolution of the 3D UTE double-echo sequence imaging is not high enough, small ligament (e.g., elbow ligament) imaging was not attempted to explore its diagnostic value. Based on the present study and associated literature, the following future research directions are planned: Further adjustment of the parameters; the performance of 3D UTE double-echo sequence imaging on the elbows of a group of volunteers; investigation into the values for observing small ligaments (as elbow ligament injury is not uncommon clinically); the performance of UTE imaging on a group of patients with early stage osteosarcoma, observing the changes to the cortical bone and periosteum in the early stages; and carrying out a quantitative study with regard to the knee meniscus and its short-T_2_ components, including dynamic enhancement and T_2_-mapping with a UTE sequence.

## Figures and Tables

**Table I:** TE range of different pulse sequence.

TE pulse sequence	TE value	Examples of pulse sequence
Very long	≥200 msec	2 DFT, HASTE, fast spin echo and EPI; very heavily long T_2_-weighted
Long	20–40 to 200 msec	2 DFT, HASTE, FlAIR, fast spin echo, EPI; heavily long T_2_-weighted
Intermediate	5–10 to 20–40 msec	2 DFT, T_1_-weighted or proton density-weighted
Short	0.5 to 5–10 msec	2 DFT, T_1_-weighted
Ultrashort	0.05–0.50 msec	Half rf pulse with radial center out sampling or radial sampling; short T_2_-weighted

TE, echo time; DFT, discrete Fourier transform; HASTE, half-Fourier acquisition single-short turbo spin-echo; EPI, echo planar imaging; T_2_, transverse relaxation time; F1AIRE, fluid attenuated inversion recovery; rf, radio frequency; T_1_, longitudinal relaxation time.

**Table II:** Ultrashort echo time pulse sequence.

Sequence Type	Acronym
Ultrashort TE	UTE
Conventional ultrashort TE	CUTE
Fat suppressed ultrashort TE	FUTE
Long T_2_ suppressed ultrashort TE	LUTE
Fat and long T_2_ suppressed ultrashort TE	EFLUTE
Short TI inversion time ultrashort TE	STUTE

T_2_, transverse relaxation time; TI, inversion time..
